# Specific TP53 mutations impair the recruitment of 53BP1 to DNA double-strand breaks underlying the mechanism of radioresistance

**DOI:** 10.1007/s00249-025-01774-8

**Published:** 2025-07-14

**Authors:** Paolo Fagherazzi, Lenka Stixová, Eva Bartova

**Affiliations:** Department of Cell Biology & Epigenetics, Institute of Biophysics of the Czech Academy of Sciences, Brno, Czech Republic

**Keywords:** TP53, 53BP1, DNA repair, Chromatin, Epigenetics

## Abstract

The tumor suppressor p53, extensively studied for over 40 years, is a key regulator of various cellular pathways, often functioning independently of its transcriptional activity. Notably, p53 has been shown to play a crucial role in DNA repair, not only in sensing DNA damage but also in influencing repair pathway choice. This work assesses the influence of p53 on the recruitment and activity of the NHEJ mediator 53BP1, focusing specifically on common p53 hotspot mutations found in human cancers. The aim is to understand how these mutations impair DNA damage response mechanisms and contribute to genetic instability, which enhances tumor survival. Analysis of p53 missense mutations (R248W, R273C, G245S) revealed mutation-specific effects on 53BP1 and RIF1 recruitment, with G245S retaining wild-type-like 53BP1 recruitment but still exhibiting enhanced BRCA1 foci formation. Given the widespread activation of NHEJ throughout the cell cycle, especially in response to radiotherapy and chemotherapy, gaining insight into how p53 mutations affect this response is vital for developing future therapeutic strategies.

**Supplementary Information:**

The online version contains supplementary material available at 10.1007/s00249-025-01774-8.

## Introduction

The tumor suppressor p53 has a central role in the response to cellular stress, including DNA damage, where it exploits the expression of genes involved in cell cycle arrest and apoptosis (Zilfou and Lowe 2009). The most detrimental DNA damage and strongest stimulus for p53 activation are DNA double-strand breaks (DSBs) deleterious lesions that can originate either from endogenous sources, like the production of reactive oxygen species (ROS), or from exogenous sources like ionizing radiation (Mehta and Haber 2014). In response to DSB formation, cells can activate two main repair pathways: the non-homologous end joining (NHEJ), directly ligating the DNA broken ends and thus active throughout the cell cycle, and the homologous recombination (HR), restoring the original information from the sister chromatid, and therefore, it is active during the S/G2 phase (Scully et al. 2019). The molecular mechanisms behind the repair pathway choice are still largely unknown, but the commitment strongly depends on central molecule hubs like p53-binding protein 1 (53BP1), which favors the recruitment of factors (such as RIF1) near DSBs and the NHEJ repair pathway choice. 53BP1 structure favors NHEJ by inhibiting DNA end resection mediated by specific nucleases and recruitment of breast cancer type 1 (BRCA1) protein that promotes HR cascade leading to error-free repair of the break (Kieffer and Lowndes 2022). 53BP1 and BRCA1 are mutually antagonists, and this interplay defines the proper timing and extent of these repair reactions in NHEJ and HR (Bunting et al. 2010). HR is generally considered error free, whereas NHEJ is typically error prone; however, exceptions to both mechanisms have been observed (Betermier et al. 2014; Guirouilh-Barbat et al. 2014).

Over the years, attention has also focused on the role of p53 in the DNA damage response (DDR), as well as in p53-induced cell cycle arrest and apoptosis. However, the notion that these are the sole tumor suppressive functions of p53 has been challenged by pivotal studies showing that certain p53 mutants, despite lacking the ability to transcriptionally regulate cell cycle and/or apoptosis, still retain tumor suppressor activity (Cosme-Blanco et al. 2007; Li et al. 2012; Liu et al. 2004; Valente et al. 2013). This broadened the view on the tumor suppressive role of the p53 protein. In the context of DSB repair, p53 has been demonstrated to interact with many factors of the main DSB repair pathways, but its role besides cell cycle arrest and apoptosis is not yet understood (Ho et al. 2019; Williams and Schumacher 2016). Previous works suggested that p53 favors the NHEJ pathway. If the p53 was absent or mutated, the recruitment of 53BP1 to the DNA damage sites was impaired without affecting the cell cycle (Moureau et al. 2016; Suchankova et al. 2017). In support of this evidence, recent work from Wang et al. illustrated how p53 can act as a sensor with fast kinetic recruitment at the UV-damaged nuclei and how this is pivotal for the downstream recruitment of 53BP1 in NHEJ repair pathway choice and keeping under control the mutagenic pathway, microhomology-mediated end joining (MMEJ), when DNA lesions are induced (Wang et al. 2022). Given that p53 is frequently mutated in a wide range of human cancers, with approximately 70% of these mutations being missense types (Sabapathy and Lane 2018), and that radiation therapy is used in over 50% of cancer patients without accounting for the molecular variations among similar tumor types (Allen et al. 2017), it is crucial to elucidate the specific molecular effects of individual p53 point mutations, particularly concerning radioresistance. Here in this work, by using a selection of p53 hotspot mutations, we found that cells bearing a p53 mutation (G245S) associated with a conformational change of the p53 protein retained the ability of 53BP1 to recognize the DNA damage site and consequently RIF1-positive foci appear in the irradiated cells. This phenomenon appeared contrary to cells carrying two specific mutations affecting the DNA-binding domain (R273C and R248W). Importantly, the ability to recruit 53BP1 and RIF1 was inversely associated with the ability to survive after treatment with different doses of ionizing radiation. Overall, these data suggest that p53 promotes the NHEJ as a first attempt to restore the breaks in DNA and that in response to DNA damage, the p53 status is a key factor determinant that unleashes the activation of mutagenic repair pathways. This reinforces the notion that different mutations are associated with different outcomes and that in future, with increased support from experimental data, p53 mutations should be considered while delineating the therapeutic strategy.

## Results

### p53 co-localizes with markers of DNA damage and accumulates at perturbed DNA regions

**Fig. 1:**
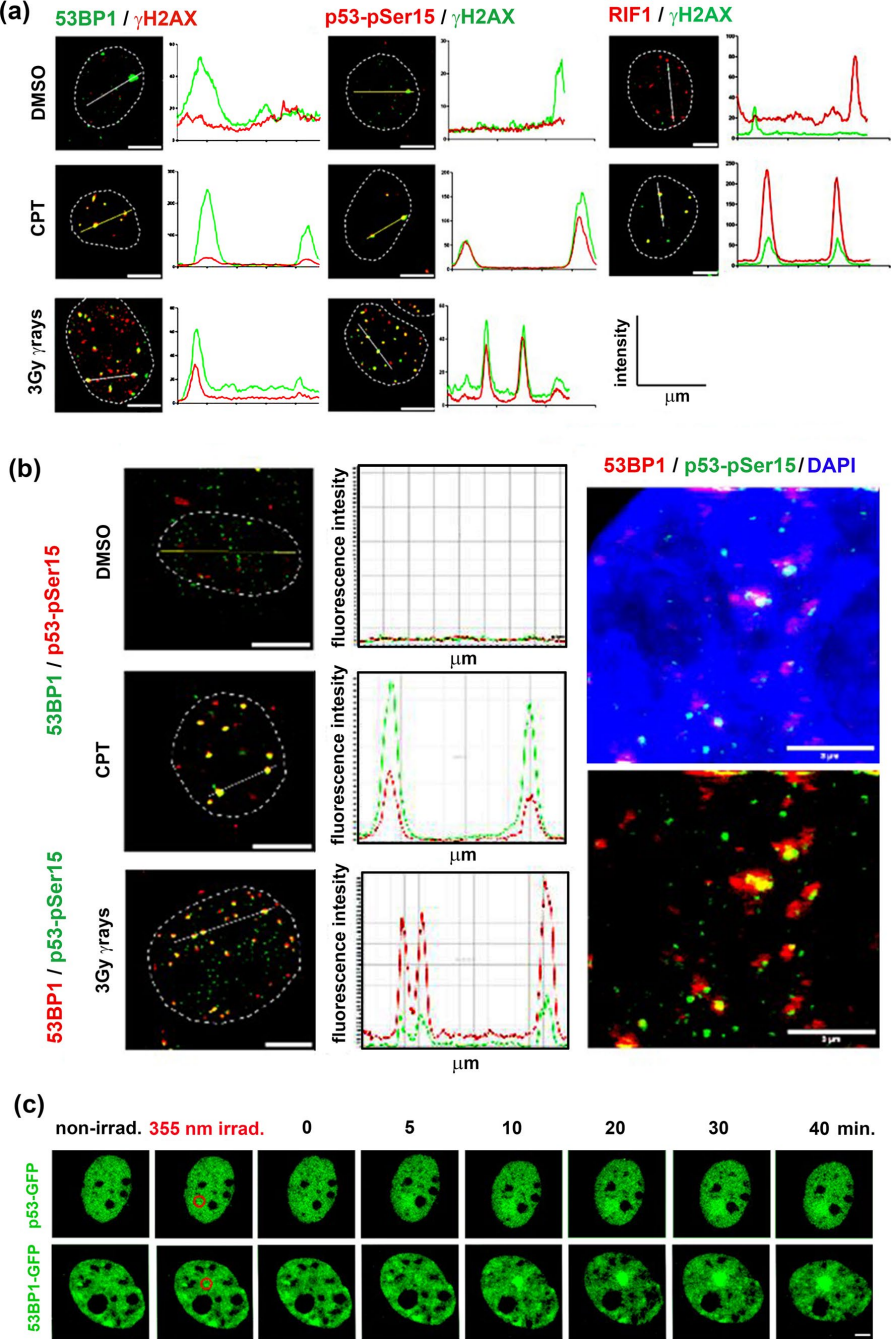
Localization and recruitment kinetic of p53 at the DNA damage sites in MCF7 cells. **a** Fluorescence intensities measured in the control condition (DMSO) and 30 min after 1 µM camptothecin (CPT) or 30 min after 3 Gy γ-rays treatments from different channels for each protein specified along the distance (µm) measured and represented with the *white line*. The graphs represent the mean fluorescence intensity (y-axis), which is expressed as a function of the distance measured inside the nuclei (distance measured in µm on the x-axis). Nucleus delimited by *dotted lines*, scale bar 7 µm. **b** On the *left*, the fluorescence intensity in MCF-7 nuclei measured for 53BP1 and phospho-Ser 15-p53 in control (DMSO), 40 min after CPT, and 40 min after 3 Gy treatment. Fluorescence intensity (y-axis in the graphs) was measured along the distance (µm) (x-axis in the graphs) represented by the *white line*. Nuclei delimited by *dotted lines*, scale bar 7 µm. On the *right*, higher magnification of a 3 Gy-irradiated MCF7 cell nucleus containing co-localizing 53BP1 and phosphor-ser15-p53 spots. The scale bar shows 3 µm. **c** Recruitment kinetics for p53-GFP and 53BP1-GFP transiently transfected in MCF7 cells. Cells were microirradiated in the regions of interest (*red dotted circle*, diameter 2 µm) with a 355 nm UV laser, and representative pictures were obtained by scanning with WLL 488 nm at the specified time points after irradiation. The scale bar indicates 8 µm

γH2AX is a well-established marker for double-strand breaks (DSBs) (Lobrich et al. 2010); therefore, we first tested whether p53, 53BP1, and its downstream factor RIF1 localize γH2AX-positive DSBs induced by γ-radiation or camptothecin. Since prior research demonstrated that serine 15 of p53 (pSer15) is a target for both ATM and DNA-PK, master kinases during DNA damage response (DDR), we took this modification as a proxy to track p53 along with the DNA damage factors (Deng et al. 1995; Nakagawa et al. 1999). As shown in Fig. 1a, the number of DNA damage foci for all monitored factors was increased in response to the ionizing radiation or camptothecin treatment. The intensity of the peaks measured within the cell nuclei confirmed that these factors formed foci specifically after DNA damage. Importantly, pSer15 p53 mirrored the trend for γH2AX, 53BP1, and RIF1 (Fig. 1a, b), suggesting its important functional role in DNA damage response. To further understand the kinetic recruitment of p53 to DNA damage sites, MCF7 cells were transiently transfected with GFP-tagged p53, and UVA-based microirradiation was used to induce DNA damage in a region of interest (Fig. 1c). This experiment confirmed the presence of p53 at the DNA damage sites and showed that accumulation of p53 occurred immediately after UVA irradiation (approximately within 5 min post-irradiation), following the recruitment of the 53BP1 protein.

To investigate the influence of p53 status in the activation of the DDR, we used non-small cell lung cancer cell lines with different p53 mutations. The p53 wild-type reference cell line containing the tetracycline-inducible system successfully expressed the wild-type TP53 gene upon 24-h induction of tetracycline (Supplementary Fig. 1a). Since p53 can oscillate its concentration in response to DNA damage, we extended our analysis to multiple points (0.5, 2, 8, and 24 h post-irradiation) to delineate early and late events in the DDR, particularly concerning 53BP1. The Western blot analysis of the p53 total pool and its activated form (pSer15) showed a general stabilization and a broad variation in the pSer15 levels in p53-mutated cell lines compared to wild type (Supplementary Fig. 1b). These observations indicate potential defects in the activation of the DDR in p53-mutated cell lines.

### Point mutations in the TP53 gene are associated with genomic instability and affect the entry to non-homologous end joining

**Fig. 2:**
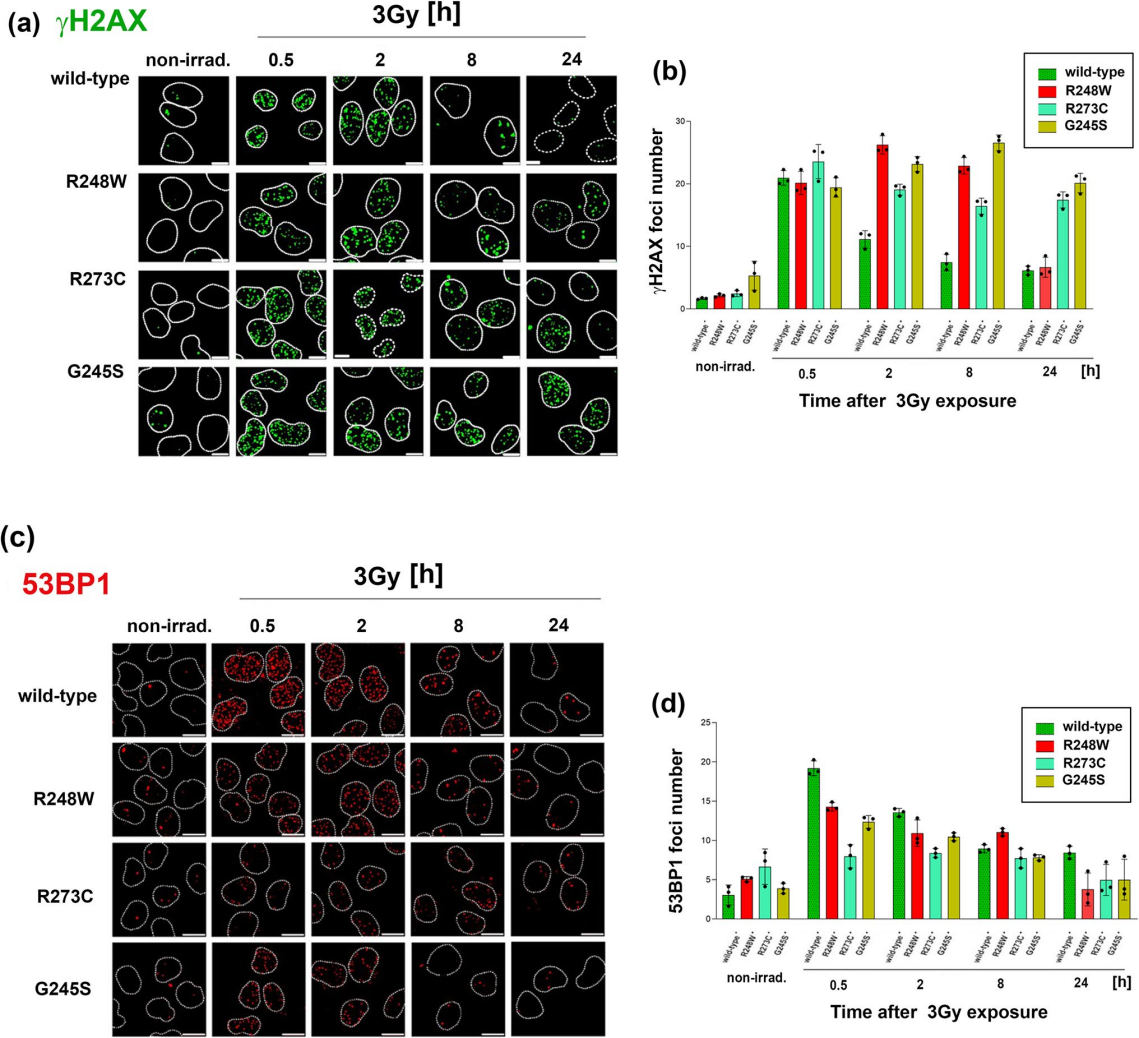
γH2AX and 53BP1 detection in 3 Gy-irradiated p53 wild-type and mutant cell lines. **a** Representative images of γH2AX foci for the cell line considered; cell nuclei are delimited by dotted lines. Scale bars 15 µm. **b** Bar chart shows the quantification of the number of foci for γH2AX per cell nucleus. **c** Representative images of 53BP1 foci, each cell nuclei are delimited by dotted lines. The scale bar shows 10 µm. **d** 53BP1 number of foci measured in each cell nucleus. The mean ± S.D. was calculated from three biological replicates (*N* > 500 cells). Statistical significance is shown in Supplementary Table 1

To investigate the impact of specific p53 point mutations on DDR dynamics, we evaluated the resolution of irradiation-induced DNA damage in mutant and wild-type cell lines. Using immunofluorescence, we quantified γH2AX foci at various time points following exposure to 3 Gy of irradiation, with the final assessment at 24 h post-irradiation to capture the steady-state resolution of DNA damage (Fig. 2a). As shown in Fig. 2b, all cell lines exhibited a uniform initial increase in γH2AX foci 30 min post-irradiation. In the p53 wild-type cell line, γH2AX foci progressively declined after the 2 h (when compared with 0.5 h after irradiation), indicative of efficient DDR activation and repair. In contrast, all p53 mutant cell lines showed a delayed or aberrant response. Notably, the R248W mutant peaked in foci count at 2 h post-irradiation and maintained elevated levels through 8 h before sharply decreasing to near wild-type levels by 24 h. Two and eight hours post-irradiation, the R273C mutant maintained a lower overall number of γH2AX foci compared to R248W and G245S but displayed significantly increased foci number when compared with R248W at 24 h. The G245S mutant cells demonstrated a continual increase in γH2AX foci, reaching a plateau at 8 h and exhibiting a marginal decrease at 24 h, but remaining significantly higher than both the wild-type and R248W mutants. The most significant disparities in γH2AX foci between wild-type and mutant lines were observed between 2 and 8 h post-irradiation, suggesting critical differences in DDR activity within this window of time. These findings suggest that mutant cell lines have impaired mechanisms for resolving DNA damage. Therefore, we next decided to track the formation of 53BP1 foci in wild-type and p53-mutated cells to understand its potential effect on the NHEJ repair pathway. As shown in Fig. 2c, d, in comparison with wild-type cells, the p53 mutant cell lines showed a lower number of 53BP1 foci formation in the early time point post-irradiation (0.5 h). Upon 8 h post-irradiation, R273C and G245S mutants exhibited foci numbers comparable to the wild type, and R248W showed a slight increase (Fig. 2d). These findings underscore the role of p53 status, as DNA-binding protein, in influencing 53BP1 (p53-binding protein 1) recruitment to DNA damage sites. While p53 does not appear to affect the formation of higher-order structures once 53BP1 foci are established, its absence or mutation disrupts the balance of repair pathway engagement, potentially contributing to aberrant DNA damage responses. Taken together, irradiation by 3 Gy of γ-rays significantly increases the number of γH2AX- and 53BP1-positive foci, as shown in Fig. 2b, d, and Supplementary Table 1, although the extent of these radiation-induced changes varies between different cell lines with specific TP53 point mutations.

### Cells carrying TP53 mutations show increased 53BP1 retention and impaired recruitment of RIF1 at DNA damage sites

**Fig. 3:**
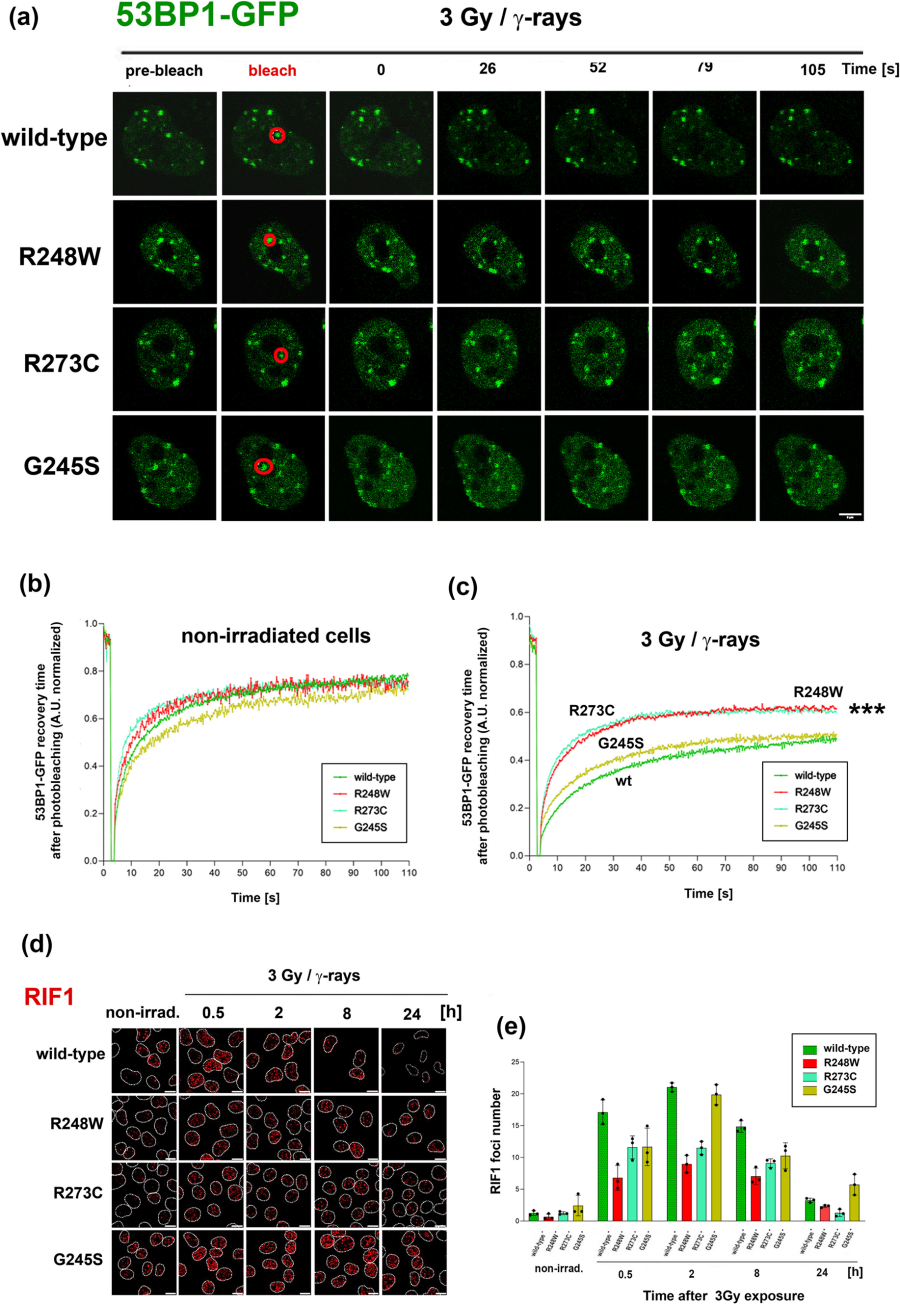
Fluorescence recovery after photobleaching for 53BP1-GFP and recruitment of RIF to DSBs. **a** Representative images of the cell lines assayed for fluorescence recovery at 1 h after 3 Gy exposure. Scale bar 5 µm. Cells were subjected to a single bleach pulse (*red circle*) followed by a real-time recording of 53BP1-GFP fluorescence recovery kinetics. The graphs represent the measurements obtained from **b** untreated cells and **c** within irradiated spots where 53BP1 accumulated. The mean ± S.E.M. is shown, *n* = 50 cells per the condition of two biologically independent experiments were analyzed, one-way ANOVA test was used; *** means *p* ≤ 0.001. **d** A representative panel of RIF1 foci. Scale bars show 15 µm. **e** Number of foci for RIF1 per cell nucleus. Bars represent the mean ± S.D. Data are from >500 cells across three biological replicates. Statistical significance is shown in Supplementary Table 1

53BP1 binding to specific epigenetic histone modifications is a prerequisite for blocking DNA end resection, enabling the recruitment of downstream factors and the formation of high-order ring-shaped structures that prevent nuclease access and facilitate non-homologous end joining (NHEJ) (Botuyan et al. 2006; Fradet-Turcotte et al. 2013; Pessina et al. 2019). To investigate potential impairments in 53BP1 binding capacity concerning p53 status, fluorescence recovery after photobleaching (FRAP) assays were performed. Cells were transiently transfected with a GFP-tagged full-length 53BP1 vector, allowed 48 h for expression, and subsequently exposed to 3 Gy of γ-ray to induce DNA damage. Live-cell imaging and FRAP analyses were conducted 1-h post-irradiation, targeting prominent GFP-tagged 53BP1 foci at DNA damage sites (Fig. 3a). In non-irradiated control cells, all samples exhibited similar rapid fluorescence recovery kinetics, indicative of high protein mobility (Fig. 3b). However, upon irradiation, substantial differences between wild-type and p53-mutated cells emerged. For instance, in comparison to the p53 wild-type and G245S mutant cells, R248W and R273C mutants demonstrated faster recovery time after photobleaching (compare recovery kinetics in Fig. 3c). These data, together with the γH2AX analysis (Fig. 2a, b), indicate alterations in the downstream response of 53BP1 during DDR in p53-mutated cell lines.

Indeed, during the activation of non-homologous end joining (NHEJ), RIF1 serves as a crucial mediator whose recruitment to DNA damage sites is partially dependent on 53BP1 and plays a key role in the formation of the Shieldin complex (Setiaputra and Durocher 2019). As shown in Fig. 3d, e and Supplementary Table 1, RIF1 foci formation was significantly impaired in p53 mutant cell lines 30 min post-irradiation, and this impairment persisted in the R248W and R273C mutants up to 8 h post-irradiation. The G245S mutant, 2 h post-irradiation, mimicked the foci number trend observed in the p53 wild-type cell line. Notably, the peak in RIF1 foci formation was observed at 2 h (Fig. 3e). Together, cells with *TP53* mutations show increased retention of 53BP1 and impaired recruitment of RIF1 at DNA damage sites, particularly in R248W and R273C mutants. These defects suggest disrupted regulation of the NHEJ pathway in the context of mutant p53.

### The homologous recombination pathway prevails in p53 mutant cells

**Fig. 4:**
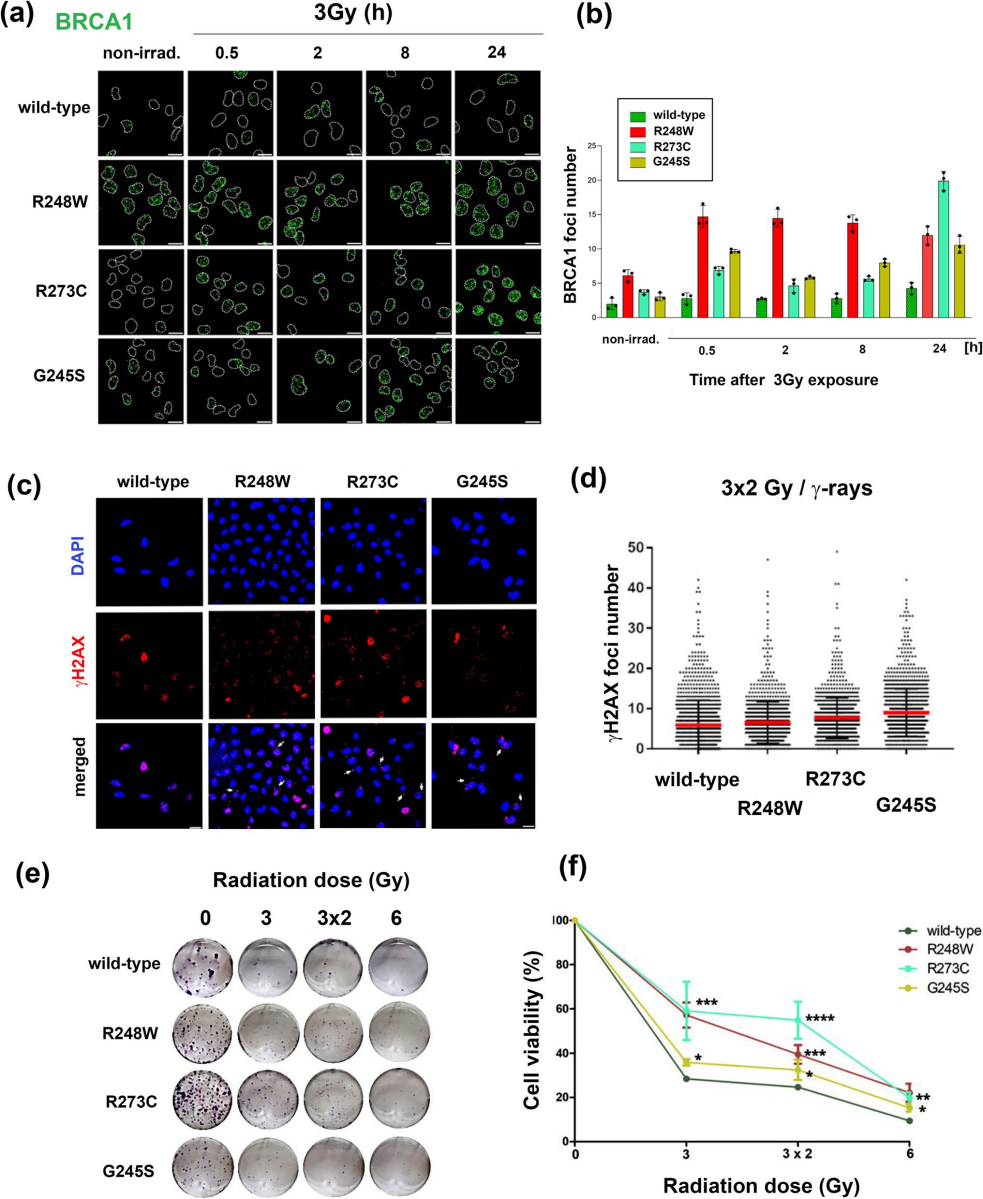
BRCA1 recruitment at the DNA damage sites, γH2AX foci formation, and survival assay in p53 wild-type and mutant cell lines exposed to a multifractionated dose of radiation (3 × 2 Gy). Panel **a** shows representative images of BRCA1 foci in non-irradiated cells and cells after 3 Gy irradiation. Each cell nucleus is delimited by dotted lines. Scale bars show 15 µm. **b** Quantification of BRCA1 foci number. *Red bars* represent the mean ± S.D. (*N* > 150 cells). **c** Immunofluorescence panel for γH2AX nuclear pattern after cellular exposition to multifractionated radiation (*white arrows* denote micronuclei-like structures). **d** Quantification of the foci number studied for γH2AX (*N* > 1000 cells). **e** Survival assay of p53 wild-type and mutant cell lines after single or multifractionated doses of radiation. **f** Quantification of the number of colonies in each cell line for each condition. Statistics were applied by one-way ANOVA with multiple comparisons (adjusted with Tukey’s correction), mean ± S.D. of two biological repetitions, *p ≤ 0.05, **p ≤ 0.01, ***p ≤ 0.001, ****p ≤ 0.0001

Prior research demonstrated that 53BP1 inhibits DNA end resection driven by specific nucleases and this in turn inhibits the homologous recombination DNA repair pathway (Adkins et al. 2013; Zgheib et al. 2009). Given the observed differences in 53BP1 recruitment to DNA damage sites among p53 mutant cell lines, it was of interest to investigate whether BRCA1, a key player in homologous recombination, could compensate for this impairment in repair. As shown in Fig. 4a, b, the quantification of BRCA1 foci number revealed higher levels in all p53 mutant cell lines following 3 Gy irradiation. Interestingly, R248W cells maintained the highest foci number, positive on BRCA1, throughout the time points. Also, both R273C and G245S mutant cell lines exhibited a significant increase in BRCA1-positive foci at 24 h post-irradiation, in comparison to their wild-type counterpart. In contrast, BRCA1 levels in p53 wild-type cells were low compared to p53 mutant cell lines, suggesting that 53BP1 effectively counteracted BRCA1 recruitment, thereby promoting the activation of NHEJ repair pathways. Along this line, the gene set enrichment analysis (GSEA) from clinical samples of patients carrying p53 mutations, obtained from The TCGA database (The Cancer Genome Atlas) (Tomczak et al. 2015), showed that genes involved in the DSB repair pathway and particularly those associated with the homologous recombination are overexpressed in these patients (Supplementary Fig. 2). Importantly, gene targets like BRCA1 and RAD51 were found to be overexpressed in these patients compared to 53BP1 and RIF1 (Supplementary Fig. 2). These findings highlight the differential engagement of DNA repair pathways in p53-mutant cells and suggest potential mechanisms of compensation for non-homologous end joining.

### Proliferation of the p53 mutant cell lines differs in response to the dose and approach of γ-radiation

Inspired by the recent work of Eke et al. and Pustovalova et al., along with the fact of limited knowledge about cell response to a multifractionated dose of radiation, we set out to investigate the response of p53-mutated cells to a multifractionated dose of radiation (Eke et al. 2020; Pustovalova et al. 2020). For this purpose, cells were exposed to 2 Gy (an amount corresponding to the daily dose administered during a single radiotherapy session) for 3 days at intervals of 24 h (3 × 2 Gy). Strikingly, γH2AX foci, even if not dramatically but still significantly, resulted in slightly higher levels in the mutant cell lines compared to the wild type (Fig. 4c, d). The highest foci numbers resulted in R273C and G245S mutant cell lines. In addition, all mutant cell lines exhibited increased micronuclei formation (white arrows in Fig. 4c) positive for both DAPI and yH2AX staining, suggesting a higher level of genome instability due to erroneous cellular divisions. The cells'ability to cope with DNA damage and genome instability varied dramatically (Fig. 4e, f). As shown in Fig. 4f, the cells bearing wild-type p53 displayed a lower survival rate compared to the rest of the cells bearing mutations, which was the minimum after exposure to a high radiation dose of 6 Gy. In general, all the p53 mutants had higher survival fractions in all conditions compared to the wild type. R273C had the highest survival fractions for both 3 Gy and 3 × 2 Gy treatments, followed by R248W mutants, which implies the highest grade of radioresistance (Fig. 4f). Overall, these data showed that aberrant DNA repair activities in p53 mutant cells resulted in the activation of HR-related DNA repair mechanisms. In general, mutations in the TP53 gene lead to genome instability promoting tumor cell survival and increasing proliferation rate.

## Discussion

The findings from this research elucidate the multifaceted role of p53 in the DNA damage response (DDR), extending beyond its well-characterized functions in apoptosis and cell cycle arrest. Based on previous work showing that phospho-p53 (ser15) co-immunoprecipitated with γH2AX from γ-irradiated MEFs (Al Rashid et al. 2005), and recent findings using proximity ligation assay to detect the interaction between p53 and γH2AX in RPE-1 cells (Wang et al. 2022), we discovered that, following camptothecin and γ-irradiation-induced DNA damage, pSer15 p53 co-localized with γH2AX as well as with 53BP1 and RIF1. These factors are crucial for preventing DNA end resection and facilitating the NHEJ repair mechanism (Di Virgilio et al. 2013; Chapman et al. 2013). Additionally, we obtained similar results as Wang et al. (2022) highlighting that p53 can be recruited at the DNA damage site with a faster kinetics than the 53BP1 protein (Wang et al. 2022). Besides the DNA target sequences associated with transcription, p53 can recognize and bind non-specific DNA sequences without epigenetic modifications, properties provided by its unstructured C-terminal domain that also allows for recruitment of DNA damage factors and is associated with NHEJ stimulatory effect in vitro (Liu and Kulesz-Martin 2001; Tang et al. 1999). Because early studies showed how the absence of p53 impacted the formation of 53BP1 foci in a cell cycle-independent way (Moureau et al. 2016), this prompted us to investigate three missense point mutations (R248W, R273C, G245S) in their ability to impact/change the activation of the DDR. We demonstrated that the recruitment of 53BP1 to chromatin after ionizing radiation varies according to the p53 mutational status, with the G245S mutation notably preserving 53BP1's association at damage sites. This suggests a mutation-specific disruption of p53’s ability to facilitate non-homologous end joining (NHEJ), as reflected by the correlated decrease in RIF1 foci formation. Whether the p53 mutations and the variability in the 53BP1 binding to DNA damage sites affected the assembly of the downstream Shieldin and CST complex, which have been described as counteractor to DNA end resection, remains to be addressed in future investigations (Dev et al. 2018; Mirman et al. 2018, 2022; Noordermeer et al. 2018). In contrast, BRCA1, a key player in homologous recombination, consistently formed foci in the irradiated cell nuclei of the p53 mutant cell lines. Interestingly, even though the G245S mutant displayed recruitment of 53BP1 that resembled those found in the p53 wild-type cells, BRCA1 foci formation was still significantly higher than in wild-type cells. This can be explained by the higher levels of BRCA1 proteins in these cells that antagonize 53BP1 and allow it to take over the residual damage sites. In support of this, the screening of patients with lung carcinoma carrying p53 missense mutations showed that BRCA1 and RAD51 genes were upregulated. Moreover, the activation of mutagenic pathways such as single-strand annealing (SSA) and microhomology-mediated end joining (MMEJ) can occur in these cells since p53 wild-type can suppress these two pathways at stalled replication forks and in response to UV irradiation or non-reparable DNA breaks (Belyaev 2010; Roy et al. 2018; Wang et al. 2022). To this fact, p53 mutants demonstrated higher survival rates following exposure to ionizing radiation compared to wild-type p53, indicating a propensity for radioresistance and potential malignant transformation linked to compromised DNA repair pathways.

In summary, our research underscores the critical and nuanced roles of the p53 protein in the DDR, positioning it as both a sensor and a modulator of the repair mechanisms employed in response to DNA damage. Future research should focus on delineating the molecular determinants of p53’s interactions with chromatin remodelers and DDR factors and exploring therapeutic strategies to target p53-mutant cancers by modulating the dynamics of repair pathways.

## Methods

### Mammalian cell culture and exposition to γ-rays

MCF-7 (p53 wild-type) and H1299 (p53 null) cell lines were grown in Dulbecco’s modified Eagle’s medium (DMEM) supplemented with 10% fetal bovine serum and 1% penicillin/streptomycin. H1299 containing R248W, R273C, and G245S point mutations of the TP53 gene was generated in Vojtesek’s laboratory (Regional Center for Applied Molecular Oncology (RECAMO) through site-directed mutagenesis and sequenced to confirm the selected mutations. H1299 expressing a tetracycline-inducible (T-Rex system, Invitrogen) wild-type form of p53 was obtained from Brazdova’s laboratory (Institute of Biophysics of the Czech Academy of Sciences), and the full expression of the protein was obtained by supplementing the medium with 1 µg/ml of tetracycline (Merck) for 24 h. To induce DNA damage, cells were exposed to different doses of γ-rays (single exposure of 3 Gy or 6 Gy, and three expositions of 2 Gy each (3 × 2 Gy) with intervals of 24 h from each exposition) using cobalt-60 (Chisostat, Chirana, Prague, Czech Republic) and harvested at the indicated time points. All cell lines were mycoplasma free and periodically monitored using a PCR-based detection kit (MycoStrip®, InvivoGen).

### Immunofluorescence staining

Cells were plated on 35 mm µ-Dish glass-bottom dishes (Ibidi, Germany) and treated as appropriate for the respective experiment. Then, the cells were fixed at the specified time points with 4% paraformaldehyde, permeabilized with 0.3% Triton X-100, and incubated with a 3% bovine serum albumin (BSA) blocking solution. The following primary antibodies were diluted in blocking solution and incubated overnight at 4 °C: anti-p53 (sc-126, Santa Cruz Biotechnologies), anti-phospho p53 (ser15) (14H61LZ4, Invitrogen), anti-RIF1 (GTX131889, Gene Tex), anti-γH2AX (05-636, Millipore), anti-γH2AX (ab2893, Abcam), anti-53BP1 (MAB3802, Millipore), anti-53BP1 (ab21083, Abcam), and anti-BRCA1 (sc-6954, Santa Cruz Biotechnologies). After incubation with the primary, cells were washed thrice with 1× PBS at room temperature and incubated with anti-rabbit Alexa fluor 594 (A11037, Thermofisher Scientific), anti-mouse Alexa fluor 647 (A21235, Thermofisher Scientific), anti-mouse Alexa fluor 488 (A21202, Thermofisher Scientific), and anti-rabbit Alexa fluor 488 (A11008, Thermofisher Scientific) secondary antibodies diluted in blocking solution, and cell nuclei were counterstained with 4′,6′-diamidino-2-phenylindole (DAPI). Images were obtained on a Leica TCS SP8 single molecule detection (SMD) confocal microscope (Leica, Manheim, Germany), equipped with a 63X/NA 1.4 oil immersion objective, using LAS AF software.

### Transfection, microirradiation, and fluorescence recovery after photobleaching (FRAP)

MCF-7 cells were transiently transfected with GFP-tagged p53 (a gift from Tyler Jacks, Addgene 12091) (Boyd et al. 2000) and GFP-tagged 53BP1 (a gift from Daniel Durocher, Addgene 60813) (Fradet-Turcotte et al. 2013) plasmids using Metafectene pro Kit (Biotex Laboratories, GimbH, Munchen, Germany) and following the manufacturer’s instructions. For the microirradiation experiments, the cells were pre-sensitized with 10 µM 5-bromo-2′ deoxyuridine (BrdU) for 24 h and microirradiated with a UVA 355 nm laser (Coherent, Inc.) connected to a Leica TCS SP5 X confocal microscope (Leica Microsystem, Manheim, Germany) within a selected region of interest (circular ROI of 2 µm diameter). The microscope was equipped with a 63X/NA 1.4 oil immersion objective, and the laser power was set at 40 mW with 100% output. FRAP experiments were performed with a Leica TCS SP8 confocal microscope (Leica, Manheim, Germany) equipped with a 63X/NA 1.4 oil immersion objective, keeping the cells inside a closed live-cell microscopy chamber at 37 °C, which was supplemented with 5% CO_2_. After the acquisition of fifteen images as pre-bleach, the bleaching was performed in the ROI with two pulses of the 488 Argon laser set at 80 mW and with 100% output. Each image frame was captured at intervals of 0.256 s within 2 min. The mobile fraction (*M*_f_) was calculated $M_{{\text{f}}} = \frac{{I_{\infty } - I_{0} }}{{I_{{\text{i}}} - I_{0} }}$, where $I_{\infty }$ is the maximal plateau value, *I*_i_ is the initial (pre-bleach) fluorescence intensity, and *I*_0_ is the minimal fluorescence intensity registered post-bleaching.

### Image correction and quantification

The analysis for the number of foci was done on ImageJ (NIH Image). Before quantifying the data, the point spread function (PSF) was calculated using the PSF generator plugin for ImageJ, which was used further to correct each image for blur through the DeconvolutionLab2 plugin on ImageJ. The quantification of the foci and fluorescence was run automatically on ImageJ by using a customized macro, adjusting the parameters for the foci mask/threshold for each dataset.

### Western blot

Whole-cell lysates were obtained by resuspending the cellular pellets in RIPA buffer (25 mM Tris–HCl pH 7.6, 150 mM NaCl, 5 mM EDTA, 1% NP40, 1% sodium deoxycholate) supplemented with 10 µg/ml PMSF, 1 µg/ml aprotinin, 1 µg/ml pepstatin A, and 20 µM NaF on ice. The supernatants were obtained after centrifugation at 13,000×*g*, and the protein concentration was determined using Bradford’s colorimetric assay. Then, protein samples were diluted in Laemmli Buffer 2X (4% SDS, 10% 2-mercaptoethanol, 20% Glycerol, 0.004% bromophenol blue, 0.125 M Tris–HCl pH 6.8), and run into pre-casted Bis–Tris 3–15% gradient gels. Proteins were then transferred to PVDF (polyvinylidene difluoride) membranes (GE Healthcare) that were subsequently blocked with 3% BSA dissolved in Tris-Buffered Saline (TBS) with the addition of 0.1% Tween-20 (TBS-T). Membranes were incubated with the anti-p53 (sc-126, Santa Cruz Biotechnologies), anti-GAPDH (sc-365062, Santa Cruz Biotechnologies), anti-phospho p53 (ser15) (14H61LZ4, Invitrogen) primary antibodies, followed by incubation with anti-rabbit (A-4914, Merck) and anti-mouse (A-9044, Merck) horseradish peroxidase-conjugated (HRP) secondary antibodies. Membranes were then captured on AmershamTM imager 680.

### Survival assay

Cells were seeded in triplicate into 6-well plates (800 cells/well). After 48 h of cultivation, the cells were exposed to the indicated doses of γ-rays. Then, cells were grown for 10 days, fixed with 4% formaldehyde, and stained with 0.1% Crystal Violet (Sigma Aldrich) dissolved into 20% ethanol. Wells were rinsed with distilled water and let dry for counting the colonies. Number of non-irradiated cells was considered as 100% (see point 0 in Fig. 4e, f).

### Analysis from the TCGA (The Cancer Genome Atlas) database

The Cancer Genome Atlas (TCGA) database was used to analyze the expression of genes associated with the DNA damage response in samples of patients with lung carcinoma carrying mutant forms of p53 and comparing them with p53 wild-type samples. A special R package TCGA biolinks (Colaprico et al. 2016) was used to filter, group, and retrieve normalized mRNA expressions. The genes considered were the following: *TP53BP1, RIF1, BRCA1,* and *RAD51*. The cluster Profiler R package was used for the Gene set enrichment analysis (GSEA), where the Gene Ontology (GO) terms and the Kyoto Encyclopedia of Genes and Genomes (KEGG) are significantly different in terms of *p* < 0.05 (Yu et al. 2012). The list of the barcodes is illustrated in the supplementary material (see Supplementary Table 2).

### Statistical analysis

The statistical analysis was run on GraphPad Prism 7 and values represent the average ± S.D. or ±S.E.M., as described in the figure legends. Statistical significance was determined using an unpaired Student's t-test or one-way ANOVA. Differences were considered statistically significant at a value of *p* < 0.05 and statistical details can be found in figure legends and Supplementary Table 1.

## Competing interests

The authors declare no competing interests.

## Supplementary Information

Below is the link to the electronic supplementary material.

Supplementary Fig. 1 Assessment of the H1299 tet-inducible system and western blot for p53 in γ-irradiated cells. (a) Representative immunofluorescence images for p53 of 3 Gy-irradiated H1299 at the indicated time points. Comparison between H1299 pre-treated with 1 µg Tet/ml for 24 h versus non-treated cells. (b) Protein levels for the total pool of p53, the phosphorylated form of p53 (pSer15), and the loading control GAPDH were detected by Western blot from whole-cell lysates collected at the specified time-points after 3 Gy exposure. Wild type = H1299 cells treated with exposure to 1 µg/ml Tet for 24 h (TIF 3519 KB)

Supplementary Fig. 2 TP53 status alters the expression programs if the main gene is involved in DSB repair. A) GSEA analysis of NSCLC clinical data samples in TP53 mutant versus wild-type (N mutants = 203, N for the wild-type = 154). B) Violin plots for the gene expression (fragment per kilobase per million = FPKM) for the selected genes on patients bearing TP53 wild type and TP53 mutated. The box plot inside the violin plots indicates the mean and the interquartile values (Each dot represents a measurement registered in a patient: graphs obtained with the R package ggplot2). The t-test was used for statistical analysis, and asterisks (****) indicate p ≤ 0.0001) (TIF 1374 KB)

Supplementary Table 1 Summary of average values and statistical analysis corresponding to Figures 2 and 3 (XLSX 36 KB)

Supplementary Table 2 Barcodes associated with patients were obtained from the TCGA database and used as specified in Supplementary Figure 2 (DOCX 33 KB)

## Data Availability

Data availability at: https://www.ibp.cz/en/research/departments/cellular-biology-and-epigenetics/open-data
