# Supplementary figures and images for: Specific TP53 mutations impair the recruitment of 53BP1 to DNA double-strand breaks underlying the mechanism of radioresistance
Source: Eur Biophys J. 2025 Jul 14;54(8):601–12. doi: 10.1007/s00249-025-01774-8 (PMC12678470; doi:10.1007/s00249-025-01774-8)

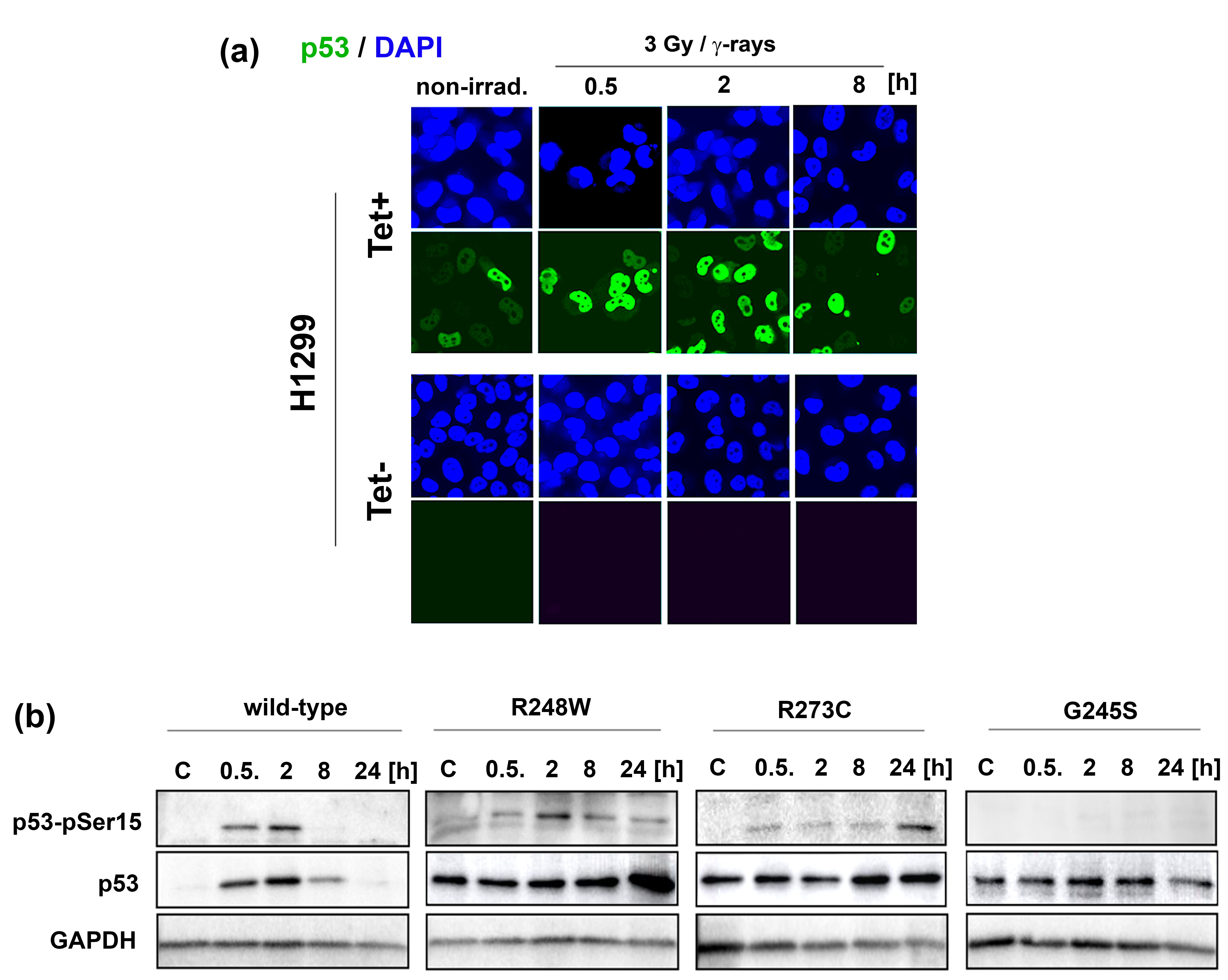

Supplement: Supplementary file 1 — Supplementary Fig. 1 Assessment of the H1299 tet-inducible system and western blot for p53 in γ-irradiated cells. (a) Representative immunofluorescence images for p53 of 3 Gy-irradiated H1299 at the indicated time points. Comparison between H1299 pre-treated with 1 µg Tet/ml for 24 h versus non-treated cells. (b) Protein levels for the total pool of p53, the phosphorylated form of p53 (pSer15), and the loading control GAPDH were detected by Western blot from whole-cell lysates collected at the specified time-points after 3 Gy exposure. Wild type = H1299 cells treated with exposure to 1 µg/ml Tet for 24 h (TIF 3519 KB) [file 249_2025_1774_MOESM1_ESM.tif]

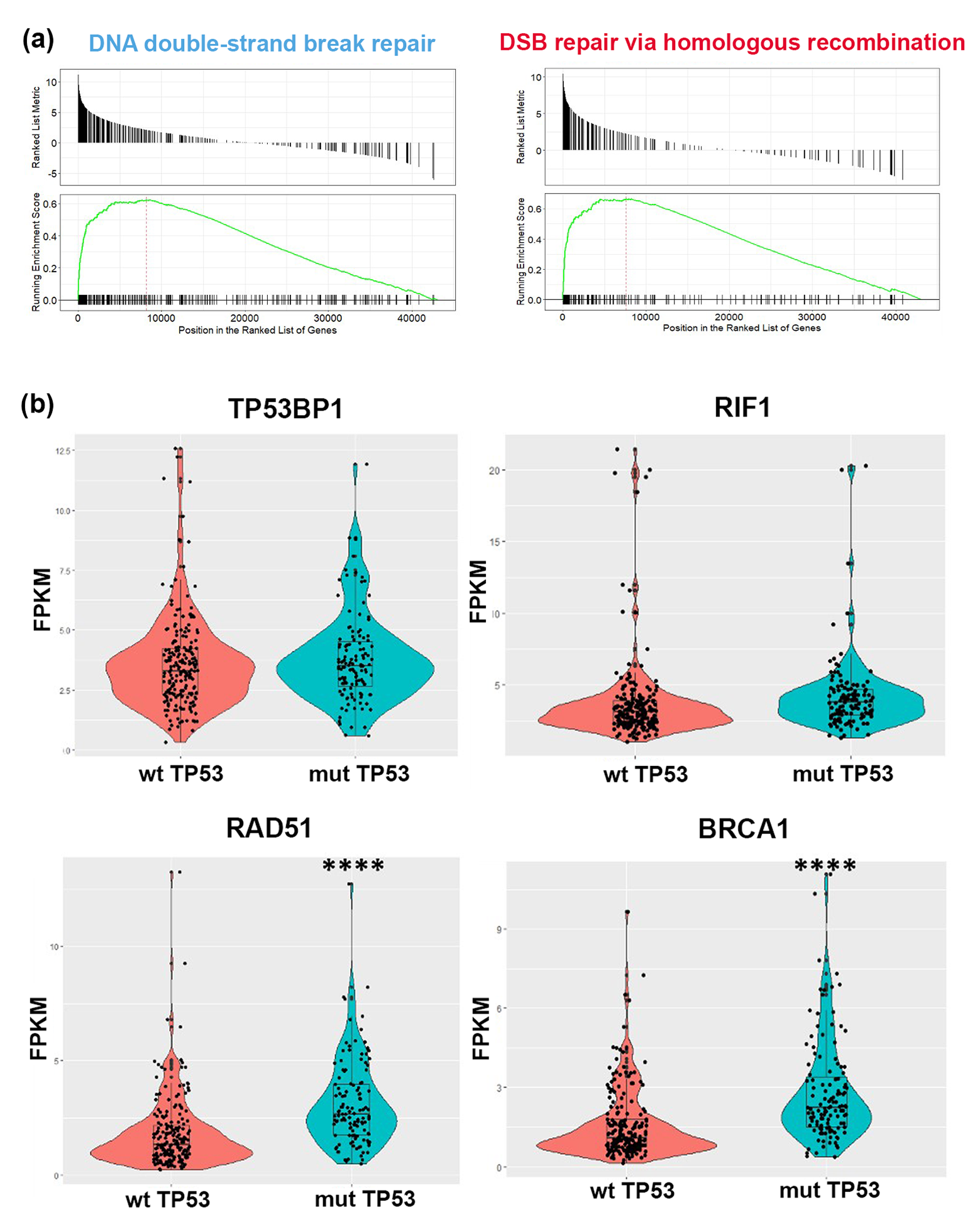

Supplement: Supplementary file 2 — Supplementary Fig. 2 TP53 status alters the expression programs if the main gene is involved in DSB repair. A) GSEA analysis of NSCLC clinical data samples in TP53 mutant versus wild-type (N mutants = 203, N for the wild-type = 154). B) Violin plots for the gene expression (fragment per kilobase per million = FPKM) for the selected genes on patients bearing TP53 wild type and TP53 mutated. The box plot inside the violin plots indicates the mean and the interquartile values (Each dot represents a measurement registered in a patient: graphs obtained with the R package ggplot2). The t-test was used for statistical analysis, and asterisks (****) indicate p ≤ 0.0001) (TIF 1374 KB) [file 249_2025_1774_MOESM2_ESM.tif]
