# Supplementary material for: Specific TP53 mutations impair the recruitment of 53BP1 to DNA double-strand breaks underlying the mechanism of radioresistance
Source: Eur Biophys J. 2025 Jul 14;54(8):601–12. doi: 10.1007/s00249-025-01774-8 (PMC12678470; doi:10.1007/s00249-025-01774-8)
Supplement: Supplementary file 4 — Supplementary Table 2 Barcodes associated with patients were obtained from the TCGA database and used as specified in Supplementary Figure 2 (DOCX 33 KB) [file 249_2025_1774_MOESM4_ESM.docx]

**Supplementary Table 2**

Barcodes associated with patients were obtained from the TCGA database and used as specified in Supplementary Figure 2.

| **Column1** | **Column2** | **Column1** | **Column2** |
| --- | --- | --- | --- |
| cases | status | cases | status |
| TCGA-J2-8194-01A-11D-2239-05 | normal TP53' | TCGA-62-A46O-01A-11D-A24I-05 | TP53 mut |
| TCGA-50-5051-01A-21D-1856-05 | normal TP53' | TCGA-69-7974-01A-11D-2185-05 | TP53 mut |
| TCGA-NJ-A55O-01A-11D-A25R-05 | normal TP53' | TCGA-O1-A52J-01A-11D-A25R-05 | TP53 mut |
| TCGA-86-8359-01A-11D-2324-05 | normal TP53' | TCGA-55-7574-01A-11D-2037-05 | TP53 mut |
| TCGA-49-AAR3-01A-11D-A411-05 | normal TP53' | TCGA-99-8033-01A-11D-2239-05 | TP53 mut |
| TCGA-62-A470-01A-11D-A24I-05 | normal TP53' | TCGA-95-7567-01A-11D-2064-05 | TP53 mut |
| TCGA-78-7537-01A-11D-2064-05 | normal TP53' | TCGA-49-4487-01A-21D-1856-05 | TP53 mut |
| TCGA-97-7941-01A-11D-2185-05 | normal TP53' | TCGA-93-A4JO-01A-21D-A24U-05 | TP53 mut |
| TCGA-55-7914-01A-11D-2168-05 | normal TP53' | TCGA-86-8280-01A-11D-2285-05 | TP53 mut |
| TCGA-62-8395-01A-11D-2324-05 | normal TP53' | TCGA-50-6593-01A-11D-1756-05 | TP53 mut |
| TCGA-78-7149-01A-11D-2037-05 | normal TP53' | TCGA-55-7726-01A-11D-2168-05 | TP53 mut |
| TCGA-97-A4M5-01A-11D-A24U-05 | normal TP53' | TCGA-44-A47G-01A-21D-A24I-05 | TP53 mut |
| TCGA-78-7153-01A-11D-2037-05 | normal TP53' | TCGA-50-8460-01A-11D-2324-05 | TP53 mut |
| TCGA-91-6835-01A-11D-1856-05 | normal TP53' | TCGA-05-4405-01A-21D-1856-05 | TP53 mut |
| TCGA-55-6975-01A-11D-1947-05 | normal TP53' | TCGA-MP-A4T9-01A-11D-A24U-05 | TP53 mut |
| TCGA-MP-A4T8-01A-11D-A24U-05 | normal TP53' | TCGA-78-7150-01A-21D-2037-05 | TP53 mut |
| TCGA-55-8299-01A-11D-2285-05 | normal TP53' | TCGA-55-8089-01A-11D-2239-05 | TP53 mut |
| TCGA-55-7724-01A-11D-2168-05 | normal TP53' | TCGA-44-7670-01A-11D-2064-05 | TP53 mut |
| TCGA-05-4384-01A-01D-1756-05 | normal TP53' | TCGA-75-6207-01A-11D-1756-05 | TP53 mut |
| TCGA-49-6761-01A-31D-1947-05 | normal TP53' | TCGA-97-7553-01A-21D-2037-05 | TP53 mut |
| TCGA-75-5146-01A-01D-1626-05 | normal TP53' | TCGA-91-6847-01A-11D-1947-05 | TP53 mut |
| TCGA-44-5643-01A-01D-1626-05 | normal TP53' | TCGA-55-7907-01A-11D-2168-05 | TP53 mut |
| TCGA-S2-AA1A-01A-12D-A398-05 | normal TP53' | TCGA-44-8117-01A-11D-2239-05 | TP53 mut |
| TCGA-62-8394-01A-11D-2324-05 | normal TP53' | TCGA-95-7947-01A-11D-2185-05 | TP53 mut |
| TCGA-MP-A4TE-01A-22D-A25R-05 | normal TP53' | TCGA-86-8056-01A-11D-2239-05 | TP53 mut |
| TCGA-95-A4VP-01A-21D-A25R-05 | normal TP53' | TCGA-53-7626-01A-12D-2064-05 | TP53 mut |
| TCGA-49-6745-01A-11D-1856-05 | normal TP53' | TCGA-49-AAR4-01A-12D-A411-05 | TP53 mut |
| TCGA-97-8171-01A-11D-2285-05 | normal TP53' | TCGA-44-5645-01A-01D-A276-05 | TP53 mut |
| TCGA-55-8097-01A-11D-2239-05 | normal TP53' | TCGA-44-5645-01B-04D-A276-05 | TP53 mut |
| TCGA-55-7903-01A-11D-2168-05 | normal TP53' | TCGA-44-5645-01A-01D-1626-05 | TP53 mut |
| TCGA-86-8278-01A-11D-2285-05 | normal TP53' | TCGA-05-4427-01A-21D-1856-05 | TP53 mut |
| TCGA-67-6217-01A-11D-1756-05 | normal TP53' | TCGA-49-4490-01A-21D-1856-05 | TP53 mut |
| TCGA-62-8398-01A-11D-2324-05 | normal TP53' | TCGA-91-6831-01A-11D-1856-05 | TP53 mut |
| TCGA-50-8457-01A-11D-2324-05 | normal TP53' | TCGA-49-AAR0-01A-21D-A398-05 | TP53 mut |
| TCGA-80-5607-01A-31D-1947-05 | normal TP53' | TCGA-44-7662-01A-11D-2064-05 | TP53 mut |
| TCGA-86-7714-01A-12D-2168-05 | normal TP53' | TCGA-55-6985-01A-11D-1947-05 | TP53 mut |
| TCGA-MP-A4TJ-01A-51D-A25R-05 | normal TP53' | TCGA-55-8096-01A-11D-2239-05 | TP53 mut |
| TCGA-50-5044-01A-21D-1856-05 | normal TP53' | TCGA-86-8074-01A-11D-2239-05 | TP53 mut |
| TCGA-55-6983-01A-11D-1947-05 | normal TP53' | TCGA-50-5049-01A-01D-1626-05 | TP53 mut |
| TCGA-95-7948-01A-11D-2185-05 | normal TP53' | TCGA-55-7573-01A-11D-2037-05 | TP53 mut |
| TCGA-78-7162-01A-21D-2064-05 | normal TP53' | TCGA-44-7660-01A-11D-2064-05 | TP53 mut |
| TCGA-55-8090-01A-11D-2239-05 | normal TP53' | TCGA-55-A4DG-01A-11D-A24I-05 | TP53 mut |
| TCGA-44-7672-01A-11D-2064-05 | normal TP53' | TCGA-55-A57B-01A-12D-A398-05 | TP53 mut |
| TCGA-64-5815-01A-01D-1626-05 | normal TP53' | TCGA-49-AARE-01A-11D-A411-05 | TP53 mut |
| TCGA-62-A46V-01A-11D-A24I-05 | normal TP53' | TCGA-55-8208-01A-11D-2239-05 | TP53 mut |
| TCGA-50-6591-01A-11D-1756-05 | normal TP53' | TCGA-44-6774-01A-21D-1856-05 | TP53 mut |
| TCGA-97-8177-01A-11D-2285-05 | normal TP53' | TCGA-55-7995-01A-11D-2185-05 | TP53 mut |
| TCGA-78-7633-01A-11D-2064-05 | normal TP53' | TCGA-MN-A4N5-01A-11D-A24U-05 | TP53 mut |
| TCGA-MP-A4SW-01A-21D-A24U-05 | normal TP53' | TCGA-64-5778-01A-01D-1626-05 | TP53 mut |
| TCGA-L9-A50W-01A-12D-A398-05 | normal TP53' | TCGA-L9-A8F4-01A-11D-A398-05 | TP53 mut |
| TCGA-J2-8192-01A-11D-2239-05 | normal TP53' | TCGA-44-6779-01A-11D-1856-05 | TP53 mut |
| TCGA-MP-A5C7-01A-11D-A25R-05 | normal TP53' | TCGA-55-8085-01A-11D-2239-05 | TP53 mut |
| TCGA-MP-A4TH-01A-31D-A25R-05 | normal TP53' | TCGA-05-5423-01A-01D-1626-05 | TP53 mut |
| TCGA-78-8655-01A-11D-2398-05 | normal TP53' | TCGA-86-7711-01A-11D-2064-05 | TP53 mut |
| TCGA-67-6215-01A-11D-1756-05 | normal TP53' | TCGA-78-7540-01A-11D-2064-05 | TP53 mut |
| TCGA-NJ-A55A-01A-11D-A25R-05 | normal TP53' | TCGA-73-4676-01A-01D-1756-05 | TP53 mut |
| TCGA-93-7348-01A-21D-2037-05 | normal TP53' | TCGA-50-6594-01A-11D-1756-05 | TP53 mut |
| TCGA-MP-A4TD-01A-32D-A25R-05 | normal TP53' | TCGA-55-7570-01A-11D-2037-05 | TP53 mut |
| TCGA-55-8615-01A-11D-2398-05 | normal TP53' | TCGA-95-8039-01A-11D-2239-05 | TP53 mut |
| TCGA-J2-A4AG-01A-11D-A24I-05 | normal TP53' | TCGA-50-5045-01A-01D-1626-05 | TP53 mut |
| TCGA-86-7953-01A-11D-2185-05 | normal TP53' | TCGA-49-AAR9-01A-21D-A411-05 | TP53 mut |
| TCGA-55-6970-01A-11D-1947-05 | normal TP53' | TCGA-97-7938-01A-11D-2168-05 | TP53 mut |
| TCGA-86-8076-01A-31D-2239-05 | normal TP53' | TCGA-83-5908-01A-21D-2285-05 | TP53 mut |
| TCGA-55-7728-01A-11D-2185-05 | normal TP53' | TCGA-55-8204-01A-11D-2239-05 | TP53 mut |
| TCGA-75-6206-01A-11D-1756-05 | normal TP53' | TCGA-05-4410-01A-21D-1856-05 | TP53 mut |
| TCGA-62-A472-01A-11D-A24I-05 | normal TP53' | TCGA-53-A4EZ-01A-12D-A24U-05 | TP53 mut |
| TCGA-50-5936-01A-11D-1626-05 | normal TP53' | TCGA-44-3917-01B-02D-A276-05 | TP53 mut |
| TCGA-55-6979-01A-11D-1947-05 | normal TP53' | TCGA-44-3917-01A-01D-A276-05 | TP53 mut |
| TCGA-50-8459-01A-11D-2324-05 | normal TP53' | TCGA-55-8616-01A-11D-2398-05 | TP53 mut |
| TCGA-55-6987-01A-11D-1947-05 | normal TP53' | TCGA-55-8510-01A-11D-2398-05 | TP53 mut |
| TCGA-97-A4M6-01A-11D-A24U-05 | normal TP53' | TCGA-44-A4SS-01A-11D-A24U-05 | TP53 mut |
| TCGA-50-6595-01A-12D-1856-05 | normal TP53' | TCGA-69-7765-01A-11D-2168-05 | TP53 mut |
| TCGA-38-7271-01A-11D-2037-05 | normal TP53' | TCGA-44-6778-01A-11D-1856-05 | TP53 mut |
| TCGA-93-7347-01A-11D-2185-05 | normal TP53' | TCGA-L9-A444-01A-21D-A24I-05 | TP53 mut |
| TCGA-50-5935-01A-11D-1756-05 | normal TP53' | TCGA-78-7155-01A-11D-2037-05 | TP53 mut |
| TCGA-44-6775-01A-11D-1856-05 | normal TP53' | TCGA-78-7146-01A-11D-2037-05 | TP53 mut |
| TCGA-44-6775-01C-02D-A276-05 | normal TP53' | TCGA-55-8514-01A-11D-2398-05 | TP53 mut |
| TCGA-44-6775-01A-11D-A276-05 | normal TP53' | TCGA-55-A493-01A-11D-A24I-05 | TP53 mut |
| TCGA-55-6642-01A-11D-1856-05 | normal TP53' | TCGA-86-A4D0-01A-11D-A24I-05 | TP53 mut |
| TCGA-50-5068-01A-01D-1626-05 | normal TP53' | TCGA-95-7043-01A-11D-1947-05 | TP53 mut |
| TCGA-05-4425-01A-01D-1756-05 | normal TP53' | TCGA-50-5939-01A-11D-1626-05 | TP53 mut |
| TCGA-55-6971-01A-11D-1947-05 | normal TP53' | TCGA-62-8402-01A-11D-2324-05 | TP53 mut |
| TCGA-86-6562-01A-11D-1756-05 | normal TP53' | TCGA-97-7937-01A-11D-2168-05 | TP53 mut |
| TCGA-50-5942-01A-21D-1756-05 | normal TP53' | TCGA-MP-A4TC-01A-11D-A24U-05 | TP53 mut |
| TCGA-97-A4M1-01A-11D-A24U-05 | normal TP53' | TCGA-55-7283-01A-11D-2037-05 | TP53 mut |
| TCGA-55-8621-01A-11D-2398-05 | normal TP53' | TCGA-55-7576-01A-11D-2064-05 | TP53 mut |
| TCGA-50-6597-01A-11D-1856-05 | normal TP53' | TCGA-49-4488-01A-01D-1756-05 | TP53 mut |
| TCGA-49-4512-01A-21D-1856-05 | normal TP53' | TCGA-95-7944-01A-11D-2185-05 | TP53 mut |
| TCGA-97-8552-01A-11D-2398-05 | normal TP53' | TCGA-05-5425-01A-02D-1626-05 | TP53 mut |
| TCGA-75-7025-01A-12D-1947-05 | normal TP53' | TCGA-64-1681-01A-11D-2064-05 | TP53 mut |
| TCGA-MP-A4T6-01A-32D-A25R-05 | normal TP53' | TCGA-69-7980-01A-11D-2185-05 | TP53 mut |
| TCGA-55-8512-01A-11D-2398-05 | normal TP53' | TCGA-NJ-A4YF-01A-12D-A25R-05 | TP53 mut |
| TCGA-50-5072-01A-21D-1856-05 | normal TP53' | TCGA-55-A48X-01A-11D-A24I-05 | TP53 mut |
| TCGA-69-7763-01A-11D-2168-05 | normal TP53' | TCGA-MN-A4N1-01A-11D-A24U-05 | TP53 mut |
| TCGA-75-6205-01A-11D-1756-05 | normal TP53' | TCGA-05-4433-01A-22D-1856-05 | TP53 mut |
| TCGA-MP-A4T2-01A-11D-A24U-05 | normal TP53' | TCGA-95-8494-01A-11D-2324-05 | TP53 mut |
| TCGA-44-2665-01B-06D-A276-05 | normal TP53' | TCGA-44-6777-01A-11D-1856-05 | TP53 mut |
| TCGA-44-2665-01A-01D-A276-05 | normal TP53' | TCGA-44-7669-01A-21D-2064-05 | TP53 mut |
| TCGA-99-AA5R-01A-11D-A398-05 | normal TP53' | TCGA-49-AARN-01A-21D-A411-05 | TP53 mut |
| TCGA-78-7160-01A-11D-2037-05 | normal TP53' | TCGA-73-A9RS-01A-11D-A411-05 | TP53 mut |
| TCGA-55-6981-01A-11D-1947-05 | normal TP53' | TCGA-L9-A5IP-01A-21D-A398-05 | TP53 mut |
| TCGA-J2-A4AE-01A-21D-A24I-05 | normal TP53' | TCGA-55-7994-01A-11D-2185-05 | TP53 mut |
| TCGA-44-2666-01B-02D-A276-05 | normal TP53' | TCGA-50-5944-01A-11D-1756-05 | TP53 mut |
| TCGA-44-2666-01A-01D-A276-05 | normal TP53' | TCGA-78-7535-01A-11D-2064-05 | TP53 mut |
| TCGA-05-5420-01A-01D-1626-05 | normal TP53' | TCGA-05-4424-01A-22D-1856-05 | TP53 mut |
| TCGA-62-8397-01A-11D-2324-05 | normal TP53' | TCGA-44-5644-01A-21D-2037-05 | TP53 mut |
| TCGA-55-6543-01A-11D-1756-05 | normal TP53' | TCGA-L9-A7SV-01A-11D-A398-05 | TP53 mut |
| TCGA-50-5932-01A-11D-1756-05 | normal TP53' | TCGA-55-7910-01A-11D-2168-05 | TP53 mut |
| TCGA-78-7167-01A-11D-2064-05 | normal TP53' | TCGA-69-7760-01A-11D-2168-05 | TP53 mut |
| TCGA-71-6725-01A-11D-1856-05 | normal TP53' | TCGA-78-8640-01A-11D-2398-05 | TP53 mut |
| TCGA-67-6216-01A-11D-1756-05 | normal TP53' | TCGA-55-A491-01A-11D-A24I-05 | TP53 mut |
| TCGA-69-7764-01A-11D-2168-05 | normal TP53' | TCGA-95-7562-01A-11D-2239-05 | TP53 mut |
| TCGA-93-A4JQ-01A-11D-A24U-05 | normal TP53' | TCGA-69-7978-01A-11D-2185-05 | TP53 mut |
| TCGA-91-8496-01A-11D-2398-05 | normal TP53' | TCGA-91-6836-01A-21D-1856-05 | TP53 mut |
| TCGA-55-7284-01B-11D-2239-05 | normal TP53' | TCGA-35-5375-01A-01D-1626-05 | TP53 mut |
| TCGA-55-6980-01A-11D-1947-05 | normal TP53' | TCGA-55-8301-01A-11D-2285-05 | TP53 mut |
| TCGA-91-A4BD-01A-11D-A24I-05 | normal TP53' | TCGA-L9-A743-01A-43D-A398-05 | TP53 mut |
| TCGA-86-8668-01A-11D-2398-05 | normal TP53' | TCGA-55-8506-01A-11D-2398-05 | TP53 mut |
| TCGA-78-7163-01A-12D-2064-05 | normal TP53' | TCGA-44-2668-01A-01D-A276-05 | TP53 mut |
| TCGA-75-6212-01A-11D-1756-05 | normal TP53' | TCGA-44-2668-01B-02D-A276-05 | TP53 mut |
| TCGA-97-7552-01A-11D-2037-05 | normal TP53' | TCGA-55-7227-01A-11D-2037-05 | TP53 mut |
| TCGA-55-8087-01A-11D-2239-05 | normal TP53' | TCGA-MP-A4TA-01A-21D-A24U-05 | TP53 mut |
| TCGA-69-7761-01A-11D-2168-05 | normal TP53' | TCGA-55-8092-01A-11D-2239-05 | TP53 mut |
| TCGA-86-A4P7-01A-11D-A24U-05 | normal TP53' | TCGA-64-1679-01A-21D-2064-05 | TP53 mut |
| TCGA-44-A47B-01A-11D-A24I-05 | normal TP53' | TCGA-86-8279-01A-11D-2285-05 | TP53 mut |
| TCGA-44-6146-01A-11D-A276-05 | normal TP53' | TCGA-62-8399-01A-21D-2324-05 | TP53 mut |
| TCGA-44-6146-01B-04D-A276-05 | normal TP53' | TCGA-49-6767-01A-11D-1856-05 | TP53 mut |
| TCGA-44-6146-01A-11D-1756-05 | normal TP53' | TCGA-75-5147-01A-01D-1626-05 | TP53 mut |
| TCGA-55-8206-01A-11D-2239-05 | normal TP53' | TCGA-55-8094-01A-11D-2239-05 | TP53 mut |
| TCGA-55-6978-01A-11D-1947-05 | normal TP53' | TCGA-49-AAQV-01A-11D-A398-05 | TP53 mut |
| TCGA-38-A44F-01A-11D-A24I-05 | normal TP53' | TCGA-86-7954-01A-11D-2185-05 | TP53 mut |
| TCGA-55-6986-01A-11D-1947-05 | normal TP53' | TCGA-78-7147-01A-11D-2037-05 | TP53 mut |
| TCGA-05-5429-01A-01D-1626-05 | normal TP53' | TCGA-44-3918-01B-02D-A276-05 | TP53 mut |
| TCGA-55-6984-01A-11D-1947-05 | normal TP53' | TCGA-44-3918-01A-01D-A276-05 | TP53 mut |
| TCGA-91-8497-01A-11D-2398-05 | normal TP53' | TCGA-73-7499-01A-11D-2185-05 | TP53 mut |
| TCGA-50-5055-01A-01D-1626-05 | normal TP53' | TCGA-91-6840-01A-11D-1947-05 | TP53 mut |
| TCGA-L4-A4E6-01A-11D-A24I-05 | normal TP53' | TCGA-62-A46R-01A-11D-A24I-05 | TP53 mut |
| TCGA-55-8619-01A-11D-2398-05 | normal TP53' | TCGA-L4-A4E5-01A-11D-A24U-05 | TP53 mut |
| TCGA-NJ-A7XG-01A-12D-A398-05 | normal TP53' | TCGA-49-AARQ-01A-11D-A411-05 | TP53 mut |
| TCGA-86-8671-01A-11D-2398-05 | normal TP53' | TCGA-78-7145-01A-11D-2037-05 | TP53 mut |
| TCGA-75-7030-01A-11D-1947-05 | normal TP53' | TCGA-86-8073-01A-11D-2239-05 | TP53 mut |
| TCGA-75-6203-01A-11D-1756-05 | normal TP53' | TCGA-93-8067-01A-11D-2285-05 | TP53 mut |
| TCGA-55-8091-01A-11D-2239-05 | normal TP53' | TCGA-53-7624-01A-11D-2064-05 | TP53 mut |
| TCGA-44-A47F-01A-11D-A24I-05 | normal TP53' | TCGA-J2-A4AD-01A-11D-A24I-05 | TP53 mut |
| TCGA-86-8358-01A-11D-2324-05 | normal TP53' | TCGA-05-5428-01A-01D-1626-05 | TP53 mut |
| TCGA-44-6148-01A-11D-1756-05 | normal TP53' | TCGA-69-8255-01A-11D-2285-05 | TP53 mut |
| TCGA-69-8453-01A-12D-2324-05 | normal TP53' | TCGA-75-5125-01A-01D-1756-05 | TP53 mut |
| TCGA-78-7143-01A-11D-2037-05 | normal TP53' | TCGA-55-7727-01A-11D-2168-05 | TP53 mut |
| TCGA-MP-A4TI-01A-21D-A24U-05 | normal TP53' | TCGA-44-6145-01A-11D-1756-05 | TP53 mut |
| TCGA-55-8505-01A-11D-2398-05 | normal TP53' | TCGA-75-6214-01A-41D-1947-05 | TP53 mut |
| TCGA-86-A4P8-01A-11D-A24U-05 | normal TP53' | TCGA-97-8179-01A-11D-2285-05 | TP53 mut |
| TCGA-97-A4M2-01A-12D-A24U-05 | normal TP53' | TCGA-86-8673-01A-11D-2398-05 | TP53 mut |
| TCGA-78-8648-01A-11D-2398-05 | normal TP53' | TCGA-55-7911-01A-11D-2168-05 | TP53 mut |
|  |  | TCGA-86-8055-01A-11D-2239-05 | TP53 mut |
|  |  | TCGA-55-8302-01A-11D-2324-05 | TP53 mut |
|  |  | TCGA-38-4632-01A-01D-1756-05 | TP53 mut |
|  |  | TCGA-49-4514-01A-21D-1856-05 | TP53 mut |
|  |  | TCGA-62-A46U-01A-11D-A24I-05 | TP53 mut |
|  |  | TCGA-MP-A4SV-01A-11D-A24U-05 | TP53 mut |
|  |  | TCGA-78-8662-01A-11D-2398-05 | TP53 mut |
|  |  | TCGA-44-8120-01A-11D-2239-05 | TP53 mut |
|  |  | TCGA-91-A4BC-01A-11D-A24I-05 | TP53 mut |
|  |  | TCGA-86-6851-01A-11D-1947-05 | TP53 mut |
|  |  | TCGA-86-8075-01A-11D-2239-05 | TP53 mut |
|  |  | TCGA-86-7701-01A-11D-2168-05 | TP53 mut |
|  |  | TCGA-MP-A4TF-01A-11D-A25R-05 | TP53 mut |
|  |  | TCGA-55-7913-01B-11D-2239-05 | TP53 mut |
|  |  | TCGA-99-8025-01A-11D-2239-05 | TP53 mut |
|  |  | TCGA-50-5933-01A-11D-1756-05 | TP53 mut |
|  |  | TCGA-95-7039-01A-11D-1947-05 | TP53 mut |
|  |  | TCGA-97-8175-01A-11D-2285-05 | TP53 mut |
|  |  | TCGA-50-6592-01A-11D-1756-05 | TP53 mut |
|  |  | TCGA-99-8028-01A-11D-2239-05 | TP53 mut |
|  |  | TCGA-78-7154-01A-11D-2037-05 | TP53 mut |
|  |  | TCGA-55-6969-01A-11D-1947-05 | TP53 mut |
|  |  | TCGA-93-A4JP-01A-11D-A24U-05 | TP53 mut |
|  |  | TCGA-05-4415-01A-22D-1856-05 | TP53 mut |
|  |  | TCGA-4B-A93V-01A-11D-A398-05 | TP53 mut |
|  |  | TCGA-38-6178-01A-11D-1756-05 | TP53 mut |
|  |  | TCGA-55-8507-01A-11D-2398-05 | TP53 mut |
|  |  | TCGA-44-7661-01A-11D-2064-05 | TP53 mut |
|  |  | TCGA-97-8547-01A-11D-2398-05 | TP53 mut |
|  |  | TCGA-44-6144-01A-11D-1756-05 | TP53 mut |
|  |  | TCGA-91-6848-01A-11D-1947-05 | TP53 mut |
|  |  | TCGA-50-6673-01A-11D-1947-05 | TP53 mut |
|  |  | TCGA-55-A48Z-01A-12D-A24U-05 | TP53 mut |
|  |  | TCGA-55-8620-01A-11D-2398-05 | TP53 mut |
|  |  | TCGA-99-7458-01A-11D-2037-05 | TP53 mut |
|  |  | TCGA-44-A4SU-01A-11D-A24U-05 | TP53 mut |
|  |  | TCGA-62-A471-01A-12D-A24I-05 | TP53 mut |
|  |  | TCGA-50-6590-01A-12D-1856-05 | TP53 mut |
|  |  | TCGA-50-5931-01A-11D-1756-05 | TP53 mut |
|  |  | TCGA-78-7220-01A-11D-2037-05 | TP53 mut |
|  |  | TCGA-44-6147-01A-11D-1756-05 | TP53 mut |
|  |  | TCGA-44-6147-01B-06D-A276-05 | TP53 mut |
|  |  | TCGA-44-6147-01A-11D-A276-05 | TP53 mut |
|  |  | TCGA-55-8614-01A-11D-2398-05 | TP53 mut |
|  |  | TCGA-78-7536-01A-11D-2064-05 | TP53 mut |
|  |  | TCGA-55-8205-01A-11D-2239-05 | TP53 mut |
|  |  | TCGA-97-7554-01A-11D-2037-05 | TP53 mut |
|  |  | TCGA-93-A4JN-01A-11D-A24U-05 | TP53 mut |
|  |  | TCGA-44-A479-01A-31D-A24I-05 | TP53 mut |
|  |  | TCGA-62-A46Y-01A-11D-A24I-05 | TP53 mut |
|  |  | TCGA-MP-A4TK-01A-11D-A24U-05 | TP53 mut |
